# Biomechanical comparison of multiple zero-profile systems in anterior cervical discectomy and fusion: a finite element analysis

**DOI:** 10.1186/s13018-025-05918-6

**Published:** 2025-05-24

**Authors:** Xiong-han Lian, Wen-jia Sun, Huo-huo Xue, Yu-fan Chen, Zhi-feng Zeng, Jing-lai Xue

**Affiliations:** 1 The School of Clinical Medicine, Fujian Medical University, Fuzhou, 350007 China; 2 Fuzhou Second General Hospital, Fuzhou, 350007 China; 3 Fujian University of Traditional Chinese Medicine, Fuzhou, 350007 China

**Keywords:** Anterior cervical discectomy and fusion, Zero-profile, Finite element, Biomechanics, Subsidence

## Abstract

**Background:**

Anterior cervical discectomy and fusion (ACDF) with zero-profile (ZP) implant is commonly used for cervical degenerative diseases, but subsidence remains a concern, particularly in osteoporosis. The two-screw ZP (TSZP), four-screw ZP (FSZP), and ROI-C implants are frequently applied, yet the biomechanical performance across varying bone qualities remains unclear.

**Methods:**

A finite element (FE) model of the cervical spine (C3-C7) was constructed with TSZP, FSZP, and ROI-C implants at C4/C5 to simulate normal and osteoporotic conditions. A 73.6 N load and 1 Nm torque were applied at C3 to simulate flexion, extension, lateral bending, and axial rotation, followed by biomechanical analysis.

**Results:**

The FSZP implant exhibited the smallest ranges of motion, followed by ROI-C, with the largest in TSZP. ROI-C showed the lowest peak implant system stresses, while TSZP had the highest on the anchoring device and FSZP on the cage. The TSZP implant had the highest cortical endplate stresses, whereas FSZP had the lowest in normal and ROI-C in osteoporosis. No significant differences were observed in adjacent intervertebral disc pressures. All parameters increased in osteoporosis, except cortical endplate stresses.

**Conclusion:**

The FSZP implant provided superior stability, while ROI-C exhibited a lower risk of implant-related complications. The TSZP implant was more prone to subsidence, which may be mitigated by optimizing stress distribution and enhancing damage prevention. Biomechanical performance was poorer under osteoporotic conditions, highlighting the need for careful surgical planning.

## Background

Anterior cervical discectomy and fusion (ACDF) was first introduced by Cloward, Smith, and Robinson in 1958 and has since been widely adopted for treating cervical degenerative diseases [1, 2]. Traditional ACDF utilizes cage-plate (CP) implants, later supplemented by zero-profile (ZP) implants. Meta-analyses have shown that ZP implants are associated with a lower incidence of postoperative dysphagia and adjacent segment degeneration compared to CP implants, leading to a recommendation for single-level ACDF [3–5]. However, ZP implants have also been linked to a higher rate of fusion device subsidence [6–8].

ACDF involves removing pathological intervertebral discs and restoring spinal stability. Unlike conventional CP implants, ZP implants lack the support of titanium plates, relying solely on interbody cages for load transmission. Furthermore, the anchoring devices of ZP implants compromise the integrity of adjacent vertebral cortical endplates during insertion, creating localized stress concentration zones that may induce microdamage and fatigue fractures. The long-term cumulative effects may lead to endplate collapse, cage subsidence, and intervertebral space narrowing. In severe cases, implant displacement, fusion failure, or neural compression may occur. Declining bone quality is a key risk factor [9, 10]. With advancements in medical technology, various ZP implants have been introduced into clinical practice, including two-screw ZP (TSZP), four-screw ZP (FSZP), and ROI-C. However, the biomechanical differences remain poorly understood.

Finite element (FE) analysis decomposes complex structures into tiny units, enabling biomechanical simulations based on fundamental physical principles. Widely used in biomedical research, this method provides valuable insights into mechanical behavior under physiological conditions. This study employs FE analysis to evaluate the biomechanical performance of different ZP implants in single-level ACDF across varying bone qualities, with implications for clinical outcomes. The findings aim to refine surgical techniques, improve treatment precision, minimize postoperative complications, and enhance patient prognosis.

## Methods

### Construction of FE model

The cervical spine imaging dataset was obtained from high-resolution thin-slice computed tomography scans of a healthy adult male volunteer (age: 52 years; height: 168 cm; weight: 70 kg; supine position; no history of cervical spine disorders). This study was approved by the Institutional Review Board of Fuzhou Second General Hospital (Ethical Approval Number: 2023001), and written informed consent was obtained before data acquisition. FE modeling was performed as follows: First, the CT dataset was imported into Mimics Medical (version 21.0; Materialise Mimics, Leuven, Belgium) for threshold-based segmentation to reconstruct the C3-C7 vertebrae. Geomagic Wrap (version 2021; Geomagic, Research Triangle Park, North Carolina, USA) was then used to refine anatomical structures and fit curved surfaces. Next, intervertebral discs, cartilage endplates, articular cartilages, and implant systems were simulated in SOLIDWORKS (version 2020; Dassault Systems SOLIDWORKS Corp, Waltham MA). The thickness of the endplate, facet joint space, and cortical bone was set to 0.5 mm [11, 12]. The intervertebral disc was subdivided into the annulus fibrosus and nucleus pulposus with a volumetric ratio of 6:4. Annulus fibers surrounded the ground substance with an inclination to the transverse plane between 15° and 30°, accounting for approximately 19% of the entire annulus fibrosus volume [13]. Finally, ligamentous complexes were simulated using nonlinear tension-only spring elements in ANSYS Workbench (version 2022 R2; ANSYS, Pennsylvania, USA) [11, 12]. Two subgroups were established based on bone condition: normal and osteoporotic. Compared to the normal group, the osteoporotic group exhibited a 33% reduction in cortical bone elastic modulus and a 66% decrease in cancellous bone elastic modulus [14, 15]. All materials were assumed to be homogeneous and isotropic, with constitutive parameters detailed in Table 1 [11, 12, 15].

**Table 1:** Spinal structure and instrumentation material properties

Spinal structure and instrumentation	Young’s modulus (MPa)	Poisson’s ratio
Cortical bone / Cortical endplate (normal)	12,000	0.30
Cortical bone / Cortical endplate (osteoporosis)	8,040	0.30
Cancellous bone (normal)	450	0.25
Cancellous bone (osteoporosis)	149	0.25
Annular fibres	110	0.30
Annulus fibrosus substance	4.2	0.49
Nucleus pulposus	1.0	0.49
Cartilaginous endplate	500	0.40
Facet joint cartilage	10.4	0.40
Anterior longitudinal ligament / Posterior	10	0.30
longitudinal ligament / Capsular ligament		
Ligamentum flavum / Interspinous ligament /	1.5	0.30
Supraspinous ligament		
Titanium	110,000	0.30
PEEK	3,600	0.30

### ACDF procedure

**Fig. 1:**
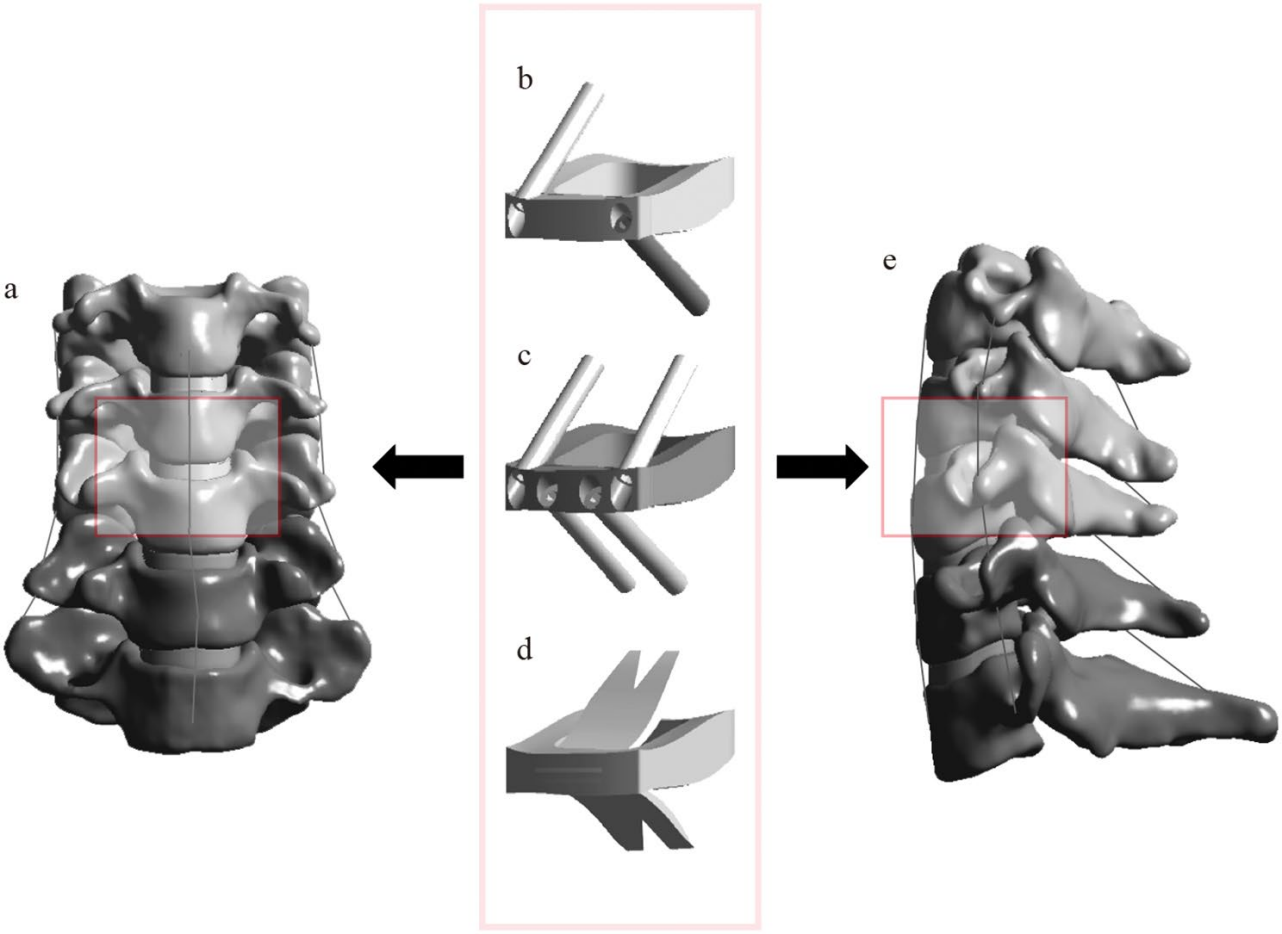
**a** Positive position of FE model; **b** TSZP implant; **c** FSZP implant; **d** ROI-C implant; **e** Lateral position of FE model

ACDF was performed at the C4/5 segment. Following adequate exposure via the anterior approach, the anterior longitudinal ligament was incised, the intervertebral disc was removed, and the disc space was prepared. Three different implant systems were respectively implanted: TSZP, FSZP, and ROI-C (Fig. 1). The TSZP implant consists of a cage and two screws, the FSZP implant includes a cage and four screws, and the ROI-C implant comprises a cage and two spacers.

### Convergence analysis

Increasing mesh density enhances accuracy but raises computational cost. Among four tested mesh sizes (2.0 mm, 1.5 mm, 1.0 mm, and 0.5 mm), a 1.0 mm mesh was selected as it balanced accuracy (peak von Mises stress variation < 5%) and computational efficiency (Table 2) [16].

**Table 2:** Convergence analysis results

Mesh size(mm)	Nodes	Units	Percentage change
2.0	234,123	112,339	>5%
1.5	360,966	156,778	>5%
1.0	778,001	381,945	<5%
0.5	3,094,412	1,682,458	<5%

### Contact, boundary, and loading conditions

**Fig. 2:**
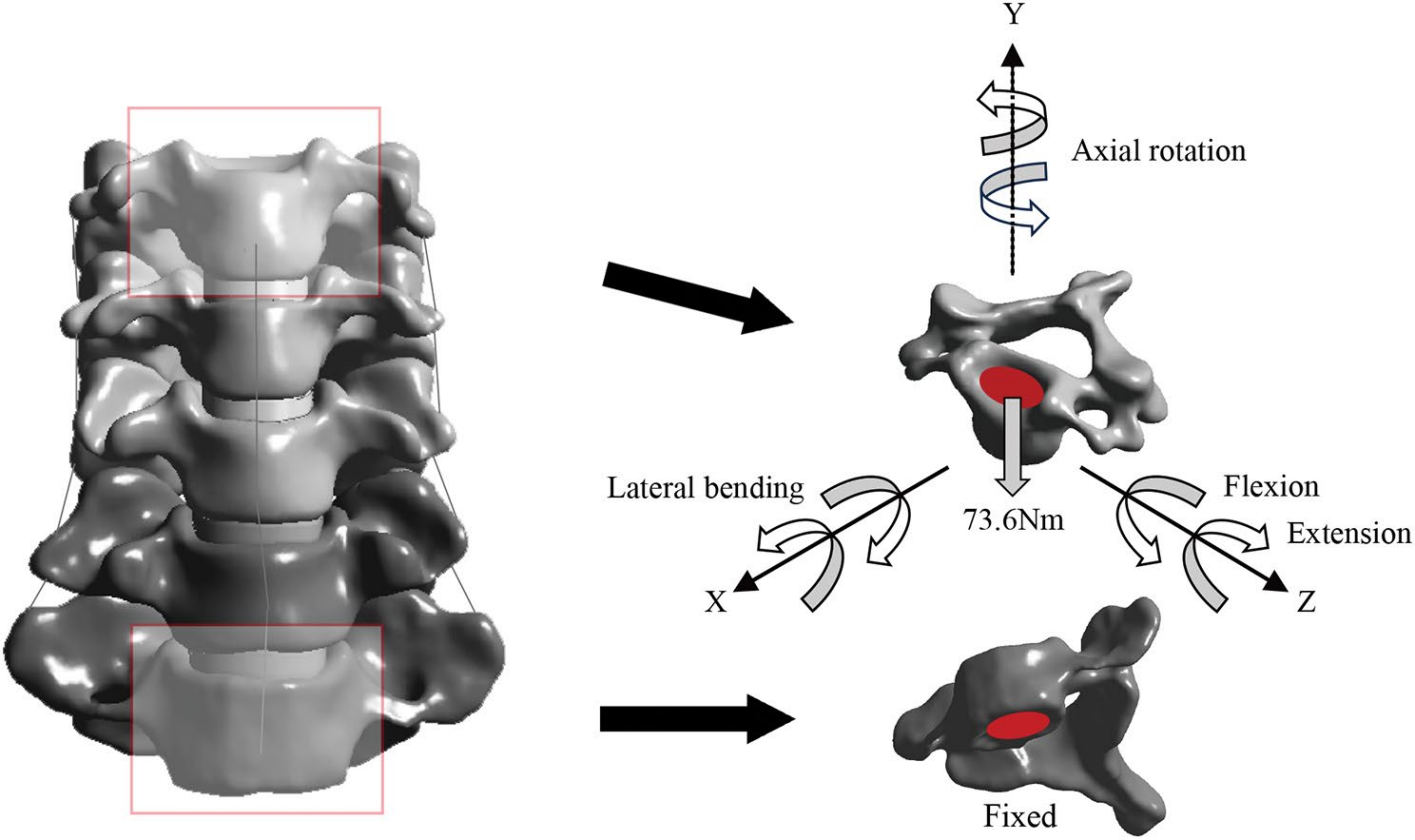
Boundary and loading conditions

Facet joint surfaces were covered with an articular cartilage layer and modeled as frictionless [13]. In all surgical models, the contacts between implant systems and anatomic structures were defined as tie to simulate rigid fusion and adequate integration [16, 17]. The inferior end of C7 was fully constrained to stabilize the FE model. A 73.6 N vertical load was applied at the superior surface of C3 to simulate head weight, combined with a 1.0 Nm torque to induce flexion, extension, lateral bending, and axial rotation (Fig. 2) [17].

## Results

### Validation of the FE model

**Fig. 3:**
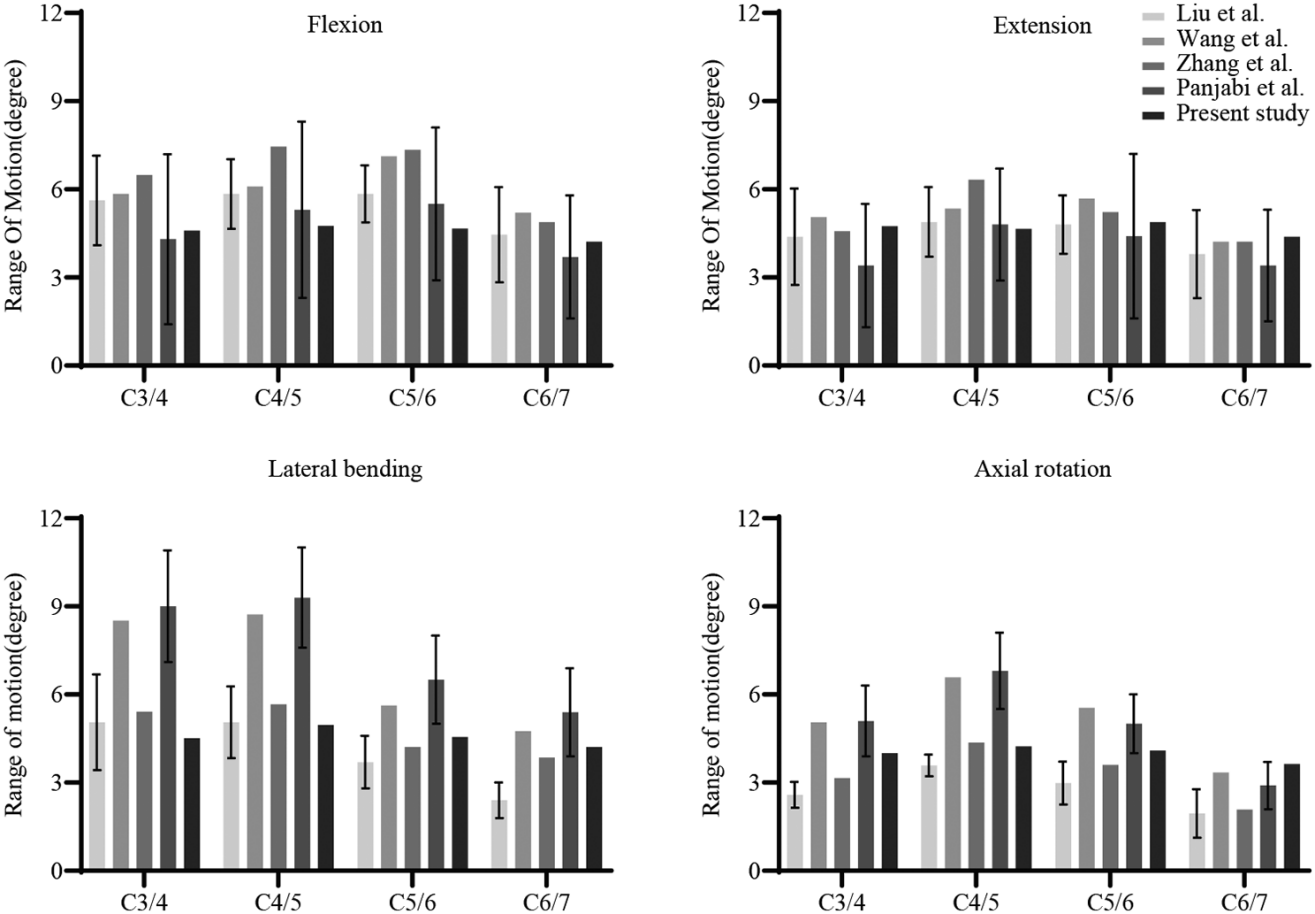
Validation of the FE Model

The FE model was validated by analyzing the ranges of motion (ROMs) under loading conditions (Fig. 3). ROMs at C3/4, C4/5, C5/6, and C6/7 during flexion were 4.59°, 4.76°, 4.66°, and 4.21°, respectively. Corresponding values were 4.74°, 4.65°, 4.88°, and 4.39° during extension; 4.52°, 4.96°, 4.55°, and 4.21° during lateral bending; and 4.01°, 4.23°, 4.09°, and 3.63° during axial rotation. These results fell within the standard deviations reported in previous studies [17–20], confirming the reliability for subsequent analysis.

### Postoperative ROM

**Fig. 4:**
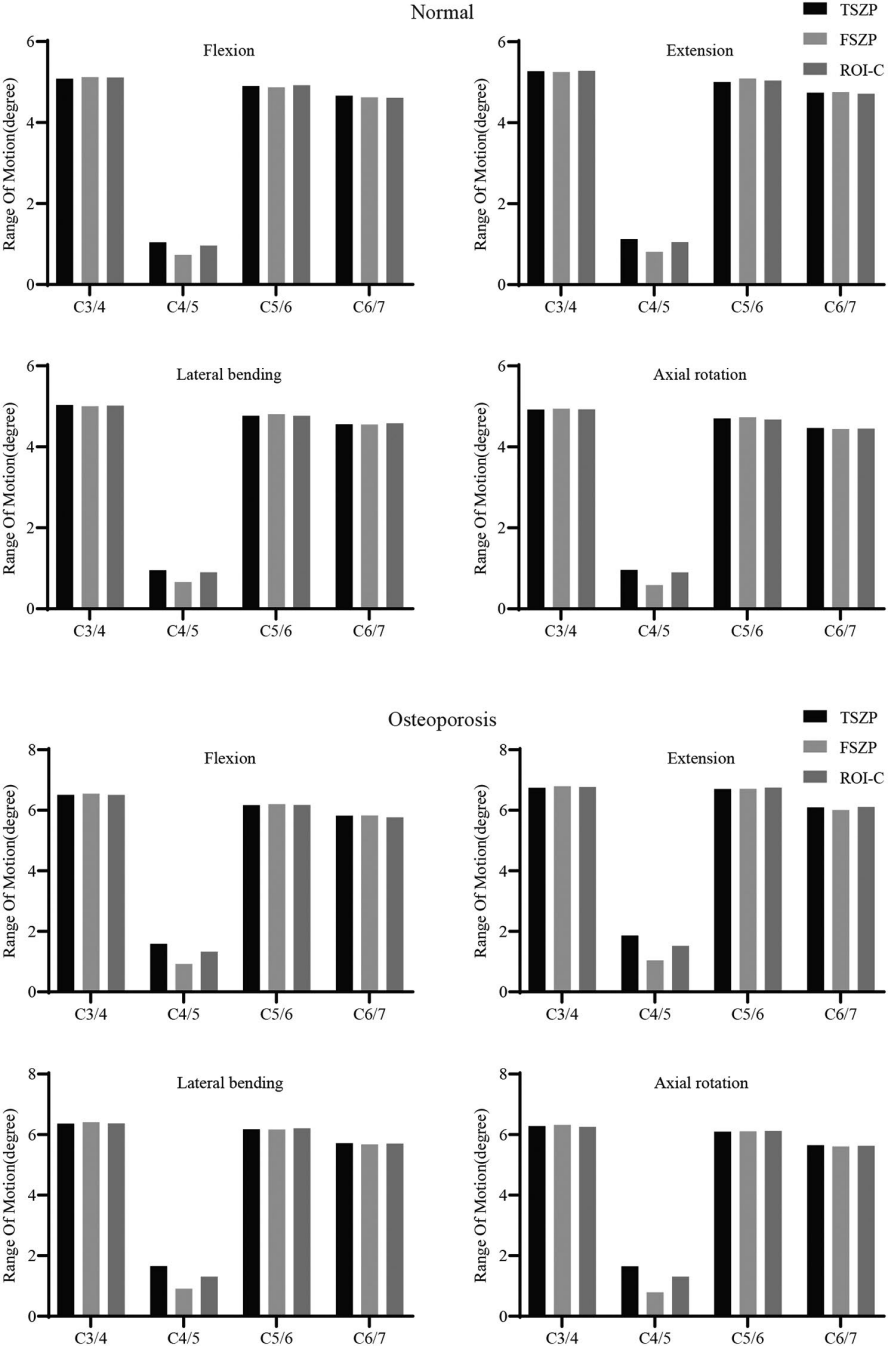
ROMs of normal group and ROMs of osteoporosis group

Postoperative ROMs are illustrated in Fig. 4. In the normal group, ROMs at C4/5 for TSZP, FSZP, and ROI-C implants were 1.04°, 0.73°, and 0.96° during flexion; 1.13°, 0.81°, and 1.05° during extension; 0.95°, 0.66°, and 0.90° during lateral bending; and 0.96°, 0.59°, and 0.90° during axial rotation. In the osteoporotic group, ROMs increased to 1.59°, 0.92°, and 1.33° during flexion; 1.86°, 1.04°, and 1.52° during extension; 1.66°, 0.91°, and 1.31° during lateral bending; and 1.65°, 0.79°, and 1.31° during axial rotation. Compared to the normal group, ROMs increased under osteoporotic conditions. FSZP implants consistently exhibited the smallest ROMs, TSZP the largest, and ROI-C intermediate values, with these differences becoming more pronounced in osteoporosis.

### Von Mises stress of implant system

**Fig. 5:**
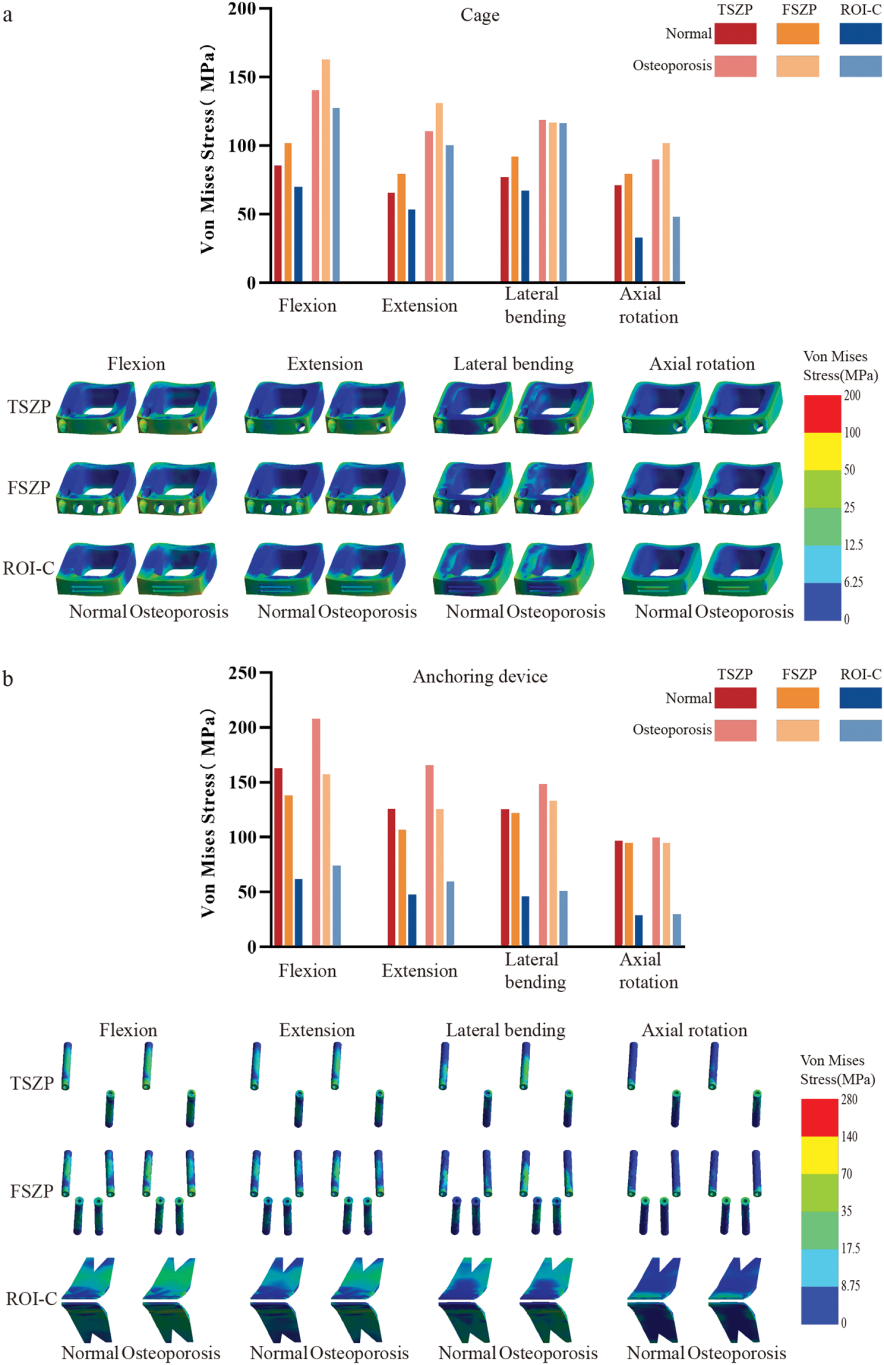
**a** Peak von Mises stress bar chart and stress distribution diagram of cages; **b** Peak von Mises stress bar chart and stress distribution diagram of anchoring devices

The stress distributions of the implant systems are shown in Fig. 5. In the normal group, the peak von Mises stresses on the cages of the TSZP, FSZP, and ROI-C implants were 85.36 MPa, 101.67 MPa, and 69.97 MPa during flexion; 65.63 MPa, 79.35 MPa, and 53.35 MPa during extension; 77.12 MPa, 91.67 MPa, and 67.05 MPa during lateral bending; and 71.28 MPa, 79.51 MPa, and 33.03 MPa during axial rotation. Under osteoporotic conditions, these values increased to 140.22 MPa, 162.70 MPa, and 127.26 MPa during flexion; 110.65 MPa, 130.87 MPa, and 100.32 MPa during extension; 118.94 MPa, 116.82 MPa, and 116.07 MPa during lateral bending; and 89.83 MPa, 101.87 MPa, and 48.16 MPa during axial rotation. In the normal group, the peak von Mises stresses on the anchoring devices of the TSZP, FSZP, and ROI-C implants were 162.71 MPa, 137.61 MPa, and 61.69 MPa during flexion; 125.66 MPa, 106.25 MPa, and 47.90 MPa during extension; 125.09 MPa, 121.69 MPa, and 45.66 MPa during lateral bending; and 96.28 MPa, 94.51 MPa, and 28.47 MPa during axial rotation. Under osteoporotic conditions, these values increased to 207.40 MPa, 157.24 MPa, and 74.07 MPa during flexion; 165.25 MPa, 125.41 MPa, and 59.57 MPa during extension; 148.23 MPa, 132.74 MPa, and 50.72 MPa during lateral bending; and 99.57 MPa, 94.17 MPa, and 29.67 MPa during axial rotation. Overall, stress levels increased under osteoporotic conditions. Among the implant systems, ROI-C exhibited the lowest stresses, FSZP had the highest cage stresses, and TSZP had the highest anchoring device stresses.

### Von Mises stress of cortical endplate

**Fig. 6:**
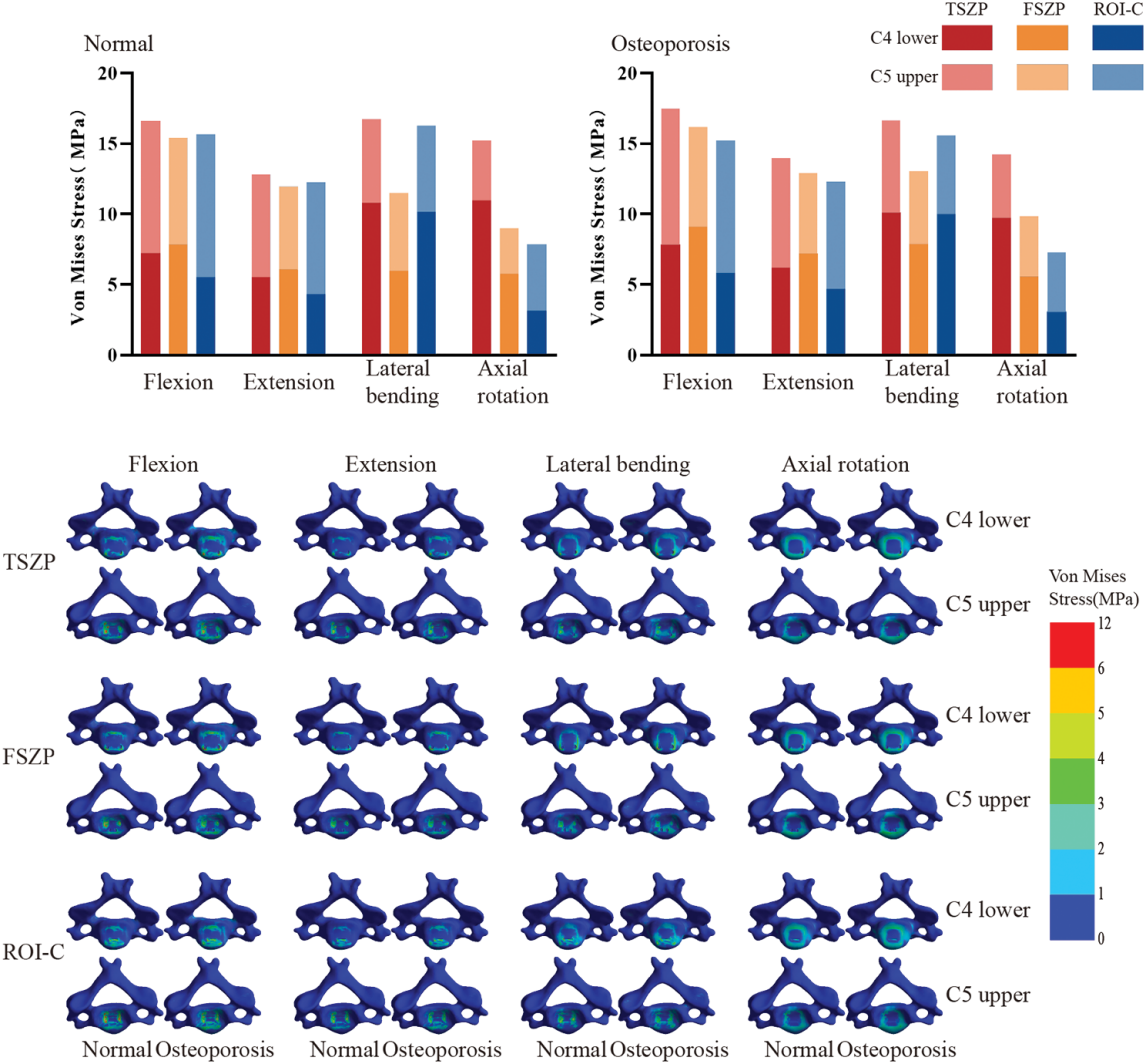
Peak von Mises stress bar chart and stress distribution diagram of adjacent cortical endplates

Cortical endplate stress distributions (Fig. 6) revealed no significant differences between the normal and osteoporotic groups. In the normal group, the total adjacent peak cortical endplate stresses for the TSZP, FSZP, and ROI-C implants were 16.58 MPa, 15.38 MPa, and 15.67 MPa during flexion; 12.81 MPa, 11.95 MPa, and 12.27 MPa during extension; 16.72 MPa, 11.47 MPa, and 16.26 MPa during lateral bending; and 15.20 MPa, 8.97 MPa, and 7.83 MPa during axial rotation. Under osteoporotic conditions, these values were 17.47 MPa, 16.16 MPa, and 15.22 MPa during flexion; 13.98 MPa, 12.88 MPa, and 12.31 MPa during extension; 16.64 MPa, 13.08 MPa, and 15.58 MPa during lateral bending; and 14.23 MPa, 9.85 MPa, and 7.29 MPa during axial rotation. Among the implant systems, TSZP exhibited the highest stresses, while FSZP had the lowest in the normal group, and ROI-C had the lowest in the osteoporotic group.

### Adjacent intervertebral disc pressure

**Fig. 7:**
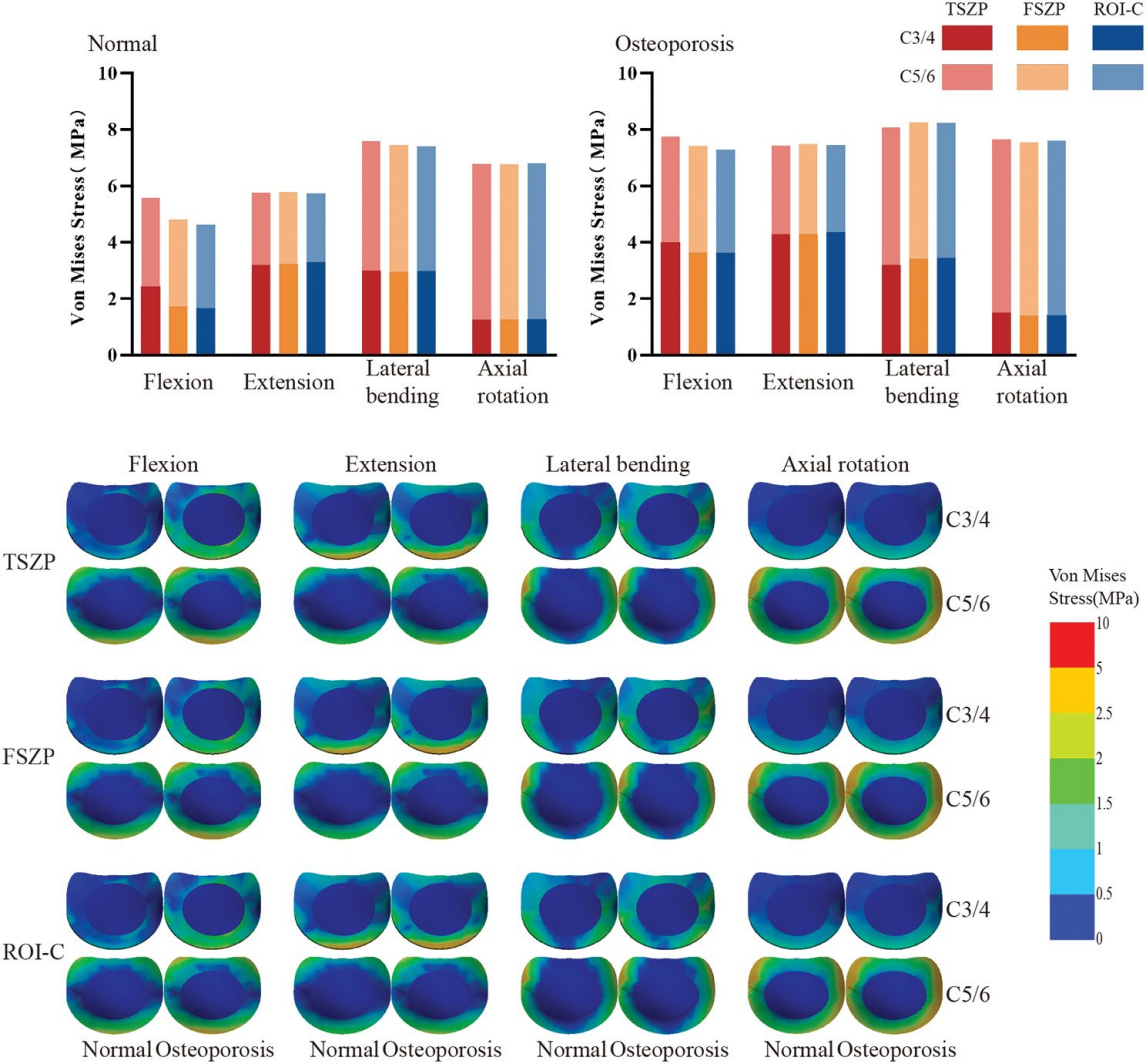
Peak von Mises stress bar chart and stress distribution diagram of adjacent IDPs

Intervertebral disc pressures (IDPs) at C3/4 and C5/6 are shown in Fig. 7. In the normal group, the total adjacent peak IDPs for the TSZP, FSZP, and ROI-C implants were 5.58 MPa, 4.81 MPa, and 4.63 MPa during flexion; 5.76 MPa, 5.78 MPa, and 5.72 MPa during extension; 7.58 MPa, 7.45 MPa, and 7.41 MPa during lateral bending; and 6.78 MPa, 6.76 MPa, and 6.80 MPa during axial rotation. Under osteoporotic conditions, these values increased to 7.75 MPa, 7.42 MPa, and 7.28 MPa during flexion; 7.44 MPa, 7.49 MPa, and 7.45 MPa during extension; 8.07 MPa, 8.24 MPa, and 8.23 MPa during lateral bending; and 7.66 MPa, 7.55 MPa, and 7.59 MPa during axial rotation. Stress levels increased under osteoporotic conditions, but differences among implant systems were minimal.

## Discussion

In this study, the C3-7 FE model was developed, including both normal and osteoporotic subgroups. The TSZP, FSZP, and ROI-C implants were placed at the C4/5 level. A comparative analysis was conducted to evaluate the ROMs, stresses on the implant systems and cortical endplates, as well as IDPs, with an exploration of the underlying biomechanical mechanisms.

### Construct stability

The key objectives of spinal surgery are adequate decompression and reconstruction of stability. Limited micromotion can facilitate bone formation and promote osseointegration [21]; however, an excessively loose implant system may result in the development of pseudoarthrosis [22]. Increased instability places greater mechanical demands on surrounding structures such as muscles, facet joints, and ligaments, which have been closely associated with postoperative axial pain and accelerated spinal degeneration.

In this study, all implant systems effectively reduced ROMs at the fusion level under various loading conditions, while compensatory increases in motion were observed in the adjacent segments. These results align with previous in vitro and FE studies [17, 23]. Among the implants, the FSZP system, which offers more anchoring points, exhibited the lowest ROMs at the C4/5 level, indicating superior stability. The ROI-C implant showed lower ROMs compared to TSZP, consistent with in vitro findings reported by Michael [24]. Postoperative ROMs were significantly higher in the osteoporotic group than in the normal group, a trend also reported by Natarajan and Li [25, 26]. In osteoporotic conditions, reductions in bone material properties diminish its load-bearing capacity, leading to greater load transfer to the implant and surrounding soft tissues. Due to the lower stiffness of ligaments, intervertebral discs, and facet joints, this increased mechanical burden results in more pronounced deformation, ultimately contributing to greater overall spinal mobility. It is also worth noting that in patients with relatively good bone quality but poor preoperative spinal stability, the FSZP implant remains a more favorable option and should be prioritized.

### Implant-related risks

Fusion devices and anchors are used in ACDF to reconstruct cervical continuity, whose peak stresses are closely associated with implant-related complications such as loosening, migration, and fracture [16, 27].

Unlike the TSZP and FSZP implants, which consist of an anterior titanium plate and a posterior PEEK interbody cage, the ROI-C implant adopts an integrated monoblock design, promoting more uniform stress distribution. The embedded spacers of the ROI-C implant help minimize stress concentrations commonly found at locking interfaces in traditional designs. By providing a larger contact area, these spacers optimize load transfer and enhance mechanical coupling at the bone-implant interface. Compared to the TSZP implant, the FSZP implant features additional anchoring points, which help distribute forces more evenly and reduce the peak stress on individual screws. Moreover, implant systems subjected to osteoporotic conditions demonstrated higher peak stresses than those in normal bone, consistent with prior findings [28–30]. This is primarily due to the compromised material properties of osteoporotic bone, which reduce its stiffness and load-bearing capacity, thereby increasing the mechanical burden on the implant. The bone-implant interface, serving as a critical zone for load transfer and a transition between materials with differing elastic moduli, is particularly vulnerable to stress concentration, potentially resulting in fixation failure under severe conditions. These findings provide valuable insights for optimizing implant design and improving biomechanical compatibility. Nevertheless, further high-quality clinical studies are necessary to validate these results and bridge the gap between computational models and clinical outcomes.

### Bone fusion

In spinal fusion surgery, internal fixation systems provide initial stability between vertebral bodies, while long-term success primarily relies on effective bone fusion. Therefore, the fusion rate is a critical clinical outcome. Adequate mechanical stress is essential for promoting bone growth; insufficient stress may result in bone resorption, whereas excessive stress can cause bone damage [27, 31]. A stress range between 2 MPa and 20 MPa is generally considered optimal for fusion [32].

Our analysis showed that all three surgical approaches produced favorable cortical endplate stress and effectively limited ROMs at the fusion segment. Clinical studies have likewise demonstrated high fusion rates across these methods, with no statistically significant differences reported [33–35]. Although our results did not show significant differences in cortical endplate stress under varying bone qualities, clinical evidence suggests that osteoporosis remains a risk factor for nonunion [36]. Further research is needed to clarify the interactions among bone quality, mechanical loading, and fusion outcomes. Additionally, stress distribution maps revealed a low-stress region at the center of the cortical endplate. Therefore, adequate bone transplantation to ensure appropriate mechanical stimulation helps improve the speed and quality of bone fusion.

### Subsidence resistance

Endplate collapse and cage subsidence are common complications of ZP systems. Clinically, they are mainly associated with excessive endplate preparation, anchoring device insertion, and low bone mineral density, which can lead to microfractures and cancellous bone exposure [9, 10, 37]. In severe cases, these complications may result in cervical malalignment, implant-related failure, and neurological deficits. Elevated cortical endplate stress is considered a key predictive factor in FE analysis [13, 16].

Stress cloud plots of the cortical endplates revealed a clear stress concentration on the side lacking screw fixation in the TSZP implant. In contrast, both the FSZP and ROI-C implants, featuring symmetrical anchoring structures, exhibited more evenly distributed and symmetrical load transfer. This suggests that anchoring devices not only help maintain the horizontal position of the interbody cage but also direct part of the stress into the vertebral body, thereby reducing the burden on the cortical endplate. In patients with normal bone quality, the anchoring devices provide strong holding strength. The FSZP implant, with more anchoring points and a larger contact area, effectively offloads the stress from the cortical endplate, resulting in the lowest peak stress among the three. However, in osteoporotic conditions, this mechanical coupling effect is weakened, and the benefits of additional anchoring points are insufficient to compensate for the bone damage caused by slotting. The dual embedded spacers of the ROI-C implant not only provide a larger contact area but also avoid repeated drilling and tapping associated with screws, making it particularly advantageous under reduced bone density.

Consistent with our previous studies [15], no significant changes in cortical endplate stress were observed with decreasing bone quality. However, due to the reduced deformation resistance of osteoporotic bone, an evident increase in strain was noted, which helps explain the promoting effect of osteoporosis on implant subsidence. Current clinical studies have not found significant differences in subsidence rates among the three implant systems [33, 35, 38]. However, future studies incorporating bone mineral density stratification are needed to further validate these findings.

### Adjacent segment degeneration

Adjacent segment disease (ASD) is a common complication following spinal fusion and, in severe cases, may require revision surgery. In this study, the three implants demonstrated no significant differences in peak IDPs, likely due to their shared ZP design. Excessive restriction of motion at the fused segment can lead to increased compensatory motion at adjacent levels. Compared to CP implants, ZP implants provide a more balanced level of stabilization, thereby mitigating excessive adjacent segment motion and potentially delaying degenerative changes. Our findings also revealed higher peak IDPs in the osteoporotic group compared to the normal group. Clinical studies by Wei and Gong have identified osteoporosis as a risk factor for ASD [39, 40]. Increased mechanical loading can subject intervertebral disc cells to both compressive and tensile stresses, which not only contribute to changes in ROM but also accelerate disc degeneration. Consistent with previous studies [13, 16], regions of high stress within the disc were primarily localized to the annulus fibrosus, which may lead to annular fissures and play a key role in the pathogenesis of ASD. Fortunately, strengthening cervical musculature may help prevent ASD by enhancing overall spinal support and reducing mechanical stress on adjacent segments [41].

### Limitation

Currently, FE analysis is widely used in biomechanics research, providing valuable insights for both basic medical science and clinical applications. However, it has several limitations. First, in this study, we modeled and analyzed the cervical spine using CT data from healthy subjects, which may not fully account for the impact of degenerative changes on the spine’s biomechanical properties. Second, our simplified cervical spine model and implant system may not accurately replicate the actual biomechanical environment. Third, most contact interactions in the FE model were defined as tied connections, potentially neglecting certain micro-movements. Finally, we focused solely on the immediate postoperative biomechanical effects, without analyzing the process or outcomes of intervertebral fusion, which may limit our understanding of the entire surgical procedure. Further investigation is also required to examine the biomechanical changes in multi-segment ZP ACDF. Therefore, the primary goal of this study is to provide trends rather than definitive conclusions.

## Conclusion

Among the three surgical methods, the FSZP implant demonstrated the best overall stability, while the ROI-C implant was associated with a lower risk of implant-related complications. In contrast, the TSZP implant exhibited inferior biomechanical performance. The three implants showed comparable performance in terms of bone fusion and adjacent segment degeneration. Optimizing stress distribution and avoiding excessive bone damage are effective strategies for preventing subsidence. Osteoporotic bone exhibited inferior performance compared to normal bone across all parameters, and surgical strategies should be carefully selected.

## Data Availability

The datasets used and analyzed during the current study are available from the corresponding author on reasonable request.

## Abbreviations

ACDF: Anterior Cervical Discectomy and Fusion
CP: Cage-Plate
ZP: Zero-Profile
TSZP: Two-Screws Zero-Profile
FSZP: Four-Screws Zero-Profile
FE: Finite Element
ROM: Range of Motion
IDP: Intervertebral Disc Pressure
ASD: Adjacent Segment Disease
